# Negative Capacitance as Universal Digital and Analog Performance Booster for Complementary MOS Transistors

**DOI:** 10.1038/s41598-019-45628-8

**Published:** 2019-06-24

**Authors:** Ali Saeidi, Farzan Jazaeri, Igor Stolichnov, Christian C. Enz, Adrian M. Ionescu

**Affiliations:** Ecole Polytechnique Federale de Lausanne, Lausanne, Switzerland

**Keywords:** Electrical and electronic engineering, Electronic devices

## Abstract

Boltzmann electron energy distribution poses a fundamental limit to lowering the energy dissipation of conventional MOS devices, a minimum increase of the gate voltage, i.e. 60 mV, is required for a 10-fold increase in drain-to-source current at 300 K. Negative Capacitance (NC) in ferroelectric materials is proposed in order to address this physical limitation of CMOS technology. A polarization destabilization in ferroelectrics causes an effective negative permittivity, resulting in a differential voltage amplification and a reduced subthreshold swing when integrated into the gate stack of a transistor. The novelty and universality of this approach relate to the fact that the gate stack is not anymore a passive part of the transistor and contributes to signal amplification. In this paper, we experimentally validate NC as a universal performance booster: (i) for complementary MOSFETs, of both n- and p-type in an advanced CMOS technology node, and, (ii) for both digital and analog significant enhancements of key figures of merit for information processing (subthreshold swing, overdrive, and current efficiency factor). Accordingly, a sub-thermal swing down to 10 mV/decade together with an enhanced current efficiency factor up to 10^5^ V^−1^ is obtained in both n- and p-type MOSFETs at room temperature by exploiting a PZT capacitor as the NC booster. As a result of the subthreshold swing reduction and overdrive improvement observed by NC, the required supply voltage to provide the same on-current is reduced by approximately 50%.

## Introduction

**Figure 1:**
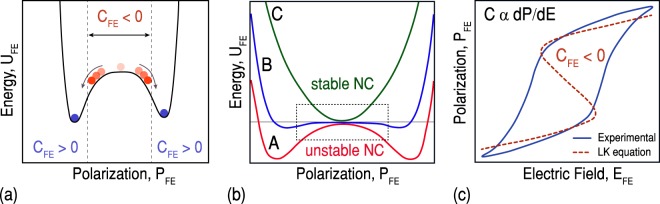
Negative capacitance in ferroelectric materials. (**a**) Energy density function of a ferroelectric capacitor in equilibrium, showing an effective NC while switching from one stable polarization state to the other one. (**b**) Ferroelectric’s NC is unstable by itself (A), but it can be partially (B) or fully stabilized (C) by placing in-series with a positive capacitor. (**c**) Measured polarization vs. electric field of a PZT film (experimental) and the fitting results of LK equation (dashed curve).

Complementary Metal-Oxide-Semiconductor (CMOS) scaling will be eventually limited by the inability to remove the heat generated in the switching process^1^. The origin of this issue can be traced back to the operation principle of the silicon CMOS devices governs by the non-scalability of thermal voltage (Boltzmann’s tyranny). This results in preventing these devices to achieve a sub-60 mV/decade subthreshold slope (SS) at room temperature. The SS of a MOSFET is obtained by

1 $$SS=\frac{\partial {V}_{g}}{\partial (log{I}_{d})}=\frac{\partial {V}_{g}}{\partial {\psi }_{s}}\times \frac{\partial {\psi }_{s}}{\partial (log{I}_{d})},$$

where *ψ*_*s*_ corresponds to the surface potential of the silicon channel. In a conventional MOSFET, the lower limit of the second term in RHS of (1) is (*k*_*B*_*T*/*q*)*Ln*(10) and cannot be any lower than 60 mV/decade at 300 K. Since V_*g*_ is linked to *ψ*_*s*_ through a capacitive voltage divider, the first term that is known as the body factor, m, is obtained as

2 $$\frac{\partial {V}_{g}}{\partial {\psi }_{s}}=1+\frac{{C}_{s}}{{C}_{MOS}},$$

exceeds one, thus limits the SS to 60 mV/decade at T = 300 K^2,3^. A sub-thermal swing can be achieved using the proposed negative capacitance (NC) of ferroelectric materials^4,5^. Negative capacitance in ferroelectrics arises from the imperfect screening of the spontaneous polarization. Imperfect screening is intrinsic to semiconductor-ferroelectric and metal-ferroelectric interfaces due to their screening lengths. The physical separation of ferroelectric bound charges from the metallic screening charges creates a depolarizing field inside the ferroelectric and destabilizes the polarization^6^. Hence, intentionally destabilizing this polarization causes an effective NC that has been proposed as a way of overcoming the fundamental limitation on the power consumption of MOSFETs^7–9^. The negative capacitance, originating from the dynamics of the stored energy in the phase transition of ferroelectric materials, results in an internal voltage amplification in an MOS device when integrated into the gate stack. The concept of NC can be understood by considering the free energy of ferroelectrics. A ferroelectric material is traditionally modeled using a double well energy landscape. The energy characteristic of a ferroelectric capacitor, depicted in Fig. 1a, is calculated by *U*_*FE*_ = *αP*^2^ + *βP*^4^ + *γP*^6^ + *E*_*ext*_.*P*, where *P* is the polarization, *E*_*ext*_ is the externally applied electric field, and *α*, *β*, and *γ* are material dependent parameters^5^. In equilibrium, the ferroelectric resides in one of the wells, providing spontaneous polarization. The capacitance of a ferroelectric material can be determined by

3 $${C}_{FE}={[\frac{{d}^{2}{U}_{FE}}{d{Q}_{FE}^{2}}]}^{-1},$$

which is positive at the wells considering the curvature of U_*FE*_ vs. Q_*FE*_ characteristic (Fig. 1a). Nevertheless, the curvature is negative around the origin (Q_*FE*_ = 0). More specifically, a ferroelectric material shows an effective NC while switching from one stable polarization state to the other one^10^. It should be remarked that NC refers to negative differential capacitance due to the small signal concept of the capacitance and relation between C_*FE*_ and U_*FE*_ (equation 3). The NC has been proven elusive for ferroelectrics in isolation and cannot be observed in experiments, exhibiting hysteretic jumps in the polarization. However, as it is qualitatively explained in Fig. 1b, if the ferroelectric placed in-series with a positive capacitor, the NC segment can be partially or fully stabilized^11,12^. This NC region can be modeled by the state-of-the-art approach for modeling the dynamics of ferroelectric capacitors relying on Landau-Khalatnikov (LK) equation, *ρ*(*dP*/*dt*) + ∇_*p*_*U*_*FE*_ = 0. Figure 1c compares the experimentally measured polarization vs. electric field of a PZT capacitor with the fitting result of the LK equation.

**Figure 2:**
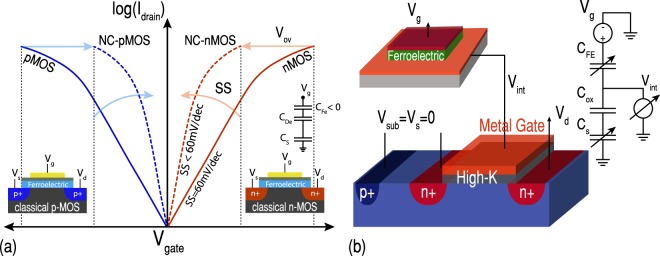
Negative capacitance MOSFET. (**a**) Performance boosting that can be achieved in both n- and p-type MOS transistors using NC of ferroelectrics in terms of SS reduction and overdrive improvement. (**b**) The employed experimental configuration of an n-type NC-FET including the capacitance model.

A ferroelectric capacitor interconnecting with the gate stack of an MOS transistor creates a series connection between C_*FE*_ and C_*MOS*_. The ferroelectric capacitor can increase the total capacitance of the gate (${C}_{total}^{-1}={C}_{FE}^{-1}+{C}_{MOS}^{-1}$) while it is stabilized in the NC region^13,14^. Specifically, the series structure brings an abrupt increase in the differential charge in the internal node (V_*int*_) by changing the gate voltage, thus providing a step-up voltage transformer^15,16^. The internal gain of NC can be defined as *β* = ∂*V*_*int*_/∂*V*_*g*_ = *C*_*FE*_/(*C*_*FE*_ + *C*_*MOS*_). Accordingly, an NC booster can provide an internal voltage amplification (*β* > 1) which results in a body factor reduction, i.e. 1/*β*, leading to the improvement of both analog and digital performances of the transistor. This effect is universal for all transistors where the gate stack contributes to the signal amplification and enhances the surface potential^17,18^. The impact of a ferroelectric gate stack on the operation of complementary MOSFETs, in terms of SS reduction and overdrive improvement, is schematically shown in Fig. 2a.

In order for NC to occur, the charge line of the baseline transistor is acquired to have an intersection with the negative slope of the polarization^10^. Otherwise, the device characteristic shows a hysteresis, corresponding to the coercive fields of the ferroelectric without performance boosting^19^. Additionally, to ensure the maximum enhancement in the non-hysteretic operation of an NC-FET, the negative value of C_*FE*_ should be well-matched with C_*MOS*_ (|*C*_*FE*_| = *C*_*MOS*_ while C_*total*_ > 0 in the whole range of operation)^16,17^. Both C_*MOS*_ and C_*FE*_ are voltage-dependent, making it extremely challenging to fully satisfy the matching condition. Therefore, the ferroelectric’s NC commonly partially gets stabilized, proposing a trade-off between the hysteretic behavior and the performance-boosting due to the NC effect. With the validity of NC concept being experimentally established^12,20–24^, it is now of paramount importance to understand the challenges involved in the design of NC-FETs, so that the steepness and the hysteresis of the device characteristic can be optimized in both n- and p-type MOSFETs. The theoretical limit for the minimum value of the SS of non-hysteretic NC-FETs has been proposed before^13^, however, a comprehensive experimental study that shows the relationship between the hysteretic behavior and steepness in negative capacitance transistors is still missing. In this regard, a PZT capacitor is fabricated for thoroughly understanding the negative capacitance concept. It is then connected to various commercial MOSFETs, fabricated in 28 nm CMOS technology node, which is demonstrated in Fig. 2b. A practical matching condition is proposed and employed to tune the hysteretic behavior of both n- and p-type NC-FETs. Afterward, the impact of NC on the performance of conventional MOSFETs is investigated by measuring and analyzing the internal node voltage. Sub-thermal swing down to 10 mV/decade is observed in n- and p-type hysteretic NC-FETs. The paper reports and discusses the trade-off between the performance boosting of NC and the hysteresis, degrading the performance by reducing the hysteretic behavior. Low hysteresis NC-FETs with subthreshold swing below 30 mV/decade are reported. The strong dependence of the NC effect on the source to drain electric field is also evidenced, reducing the impact by increasing the absolute value of V_*ds*_. It is also experimentally validated that a poly-domain ferroelectric capacitor in steady states cannot have more than one stable NC domain at the time, showing a different polarization characteristic from the expected S-shape of a single-domain ferroelectric. The reported performance improvements in this work is limited to the static device characteristics. The proposed experimental method of this study, which has been employed in order to study different matching conditions, may affect the dynamic performance and frequency response of transistors, which cannot be investigated by this method. Therefore, no frequency measurement has been carried out in this study. However, in a fully integrated NC-FET, using a sufficiently fast switching ferroelectric such as PZT, it is expected to have the NC as an effective performance booster of CMOS even in high frequencies.

## n-type Negative Capacitance MOSFETs

**Figure 3:**
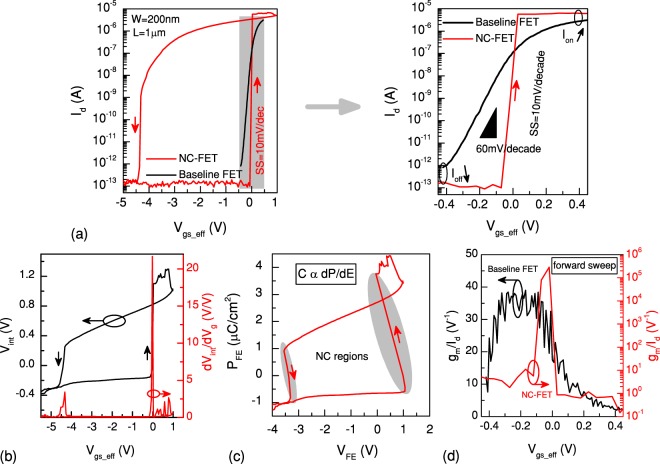
Hysteretic n-type NC-FET. (**a**) Transfer characteristic shows a super steep transition of 10 mV/decade together with a hysteresis of 4.5 V (V_*ds*_ = 100 mV). (**b**) A remarkable amplification (defined as dV_*int*_/dV_*g*_) up to 20 V/V is achieved in the regions corresponding to the negative slope of the polarization (**c**). Extracted current efficiency factor of the device represents a significant boosting, up to 10^5^ V^−1^ (**d**).

Figure 3a illustrates the input transfer characteristic of an n-type NC-FET where the gate of a baseline FET (W = 200 nm, L = 1 *μ*m) is loaded with a PZT capacitor having an area of 30 × 30 *μm*^2^. The gate voltage is swept from −3 V to +3 V and back to −3 V while the drain voltage is set to 0.1 V. In order to decouple the impact of the threshold voltage variation, curves are plotted with respect to the effective gate voltage, i.e. V _*gs*_*eff*_ = V_*gs*_ − V_*th*_. This makes the results comparable for different values of the threshold voltage. With the aid of an internal electrode, a step-up conversion of the internal voltage is explicitly observed as a result of the ionic movement in PZT. To qualitatively determine the voltage gain, dV_*int*_/dV_*g*_ vs. V_*gs*_*eff*_ is calculated, representing a significant amplification up to 20 V/V (Fig. 3b). This internal voltage increase allows the surface potential to be higher than the gate voltage, leading to a body factor below 1. Therefore, an SS of 10 mV/decade is observed over seven orders of magnitude of the drain current which is the widest operation range of NC ever reported. The overdrive voltage is also improved by 50% (0.45 V). Using the internal electrode and imposing the displacement vector continuity, a negative slope of the polarization is extracted in a certain range of the polarization. This corresponds to the subthreshold region where a significant boosting of performance is reached (Fig. 3c). An effective NC during a wide range of the gate voltage during the forward sweep leads to a significant voltage amplification (peak of 20 V/V). It should be noted that due to the charge balance conditions, only a small fraction of the polarization get switched^25^ and the results are obtained based on the minor loops. A remarkable enhancement of the current efficiency factor, g_*m*_/I_*d*_, with a peak of 10^5^ V^−1^, is demonstrated when the device is operating in the weak-inversion regime (Fig. 3d). A significant improvement of both digital and analog FoM of the reference MOSFET is realized due to the NC effect of the PZT capacitor. The huge gain of NC, resulting in the super steep switching feature, is accompanied by a large hysteresis of 4.5 V as a trade-off^17,26^. This is attributed to the second term of the RHS of the LK equation (the third term has a negligible effect), which causes a non-linearity. Hence, to implement NC switches without hysteresis, the ferroelectric and transistor parameters should be chosen wisely to maximize the steepness as well as minimizing the hysteresis^17^.

The undesirable hysteretic operation of NC-FETs can be alleviated with a better matching of the ferroelectric and MOS capacitances which ensures the *C*_*total*_ > 0 stability condition in a wide range of the applied gate voltage^27^. Considering ${C}_{total}^{-1}={C}_{FE}^{-1}+{C}_{ox}^{-1}+{C}_{si}^{-1}$, where *C*_*ox*_ and *C*_*si*_ correspond to the gate linear dielectric and silicon capacitances, the stability condition can be written in a practical way as follows

4 $$(\frac{{S}_{gate}}{{S}_{FE}}) < \frac{5\gamma }{\mathrm{4(3}{\beta }^{2}-5\alpha \gamma )}(\frac{1}{{d}_{FE}})[\frac{{d}_{ox}}{{\varepsilon }_{Si{O}_{2}}}+\frac{{d}_{si}}{{\varepsilon }_{si}}]\mathrm{.}$$

In equation (6), d, S, and *ε* are the thickness, area, and the permittivity of the corresponding layer, respectively.

**Figure 4:**
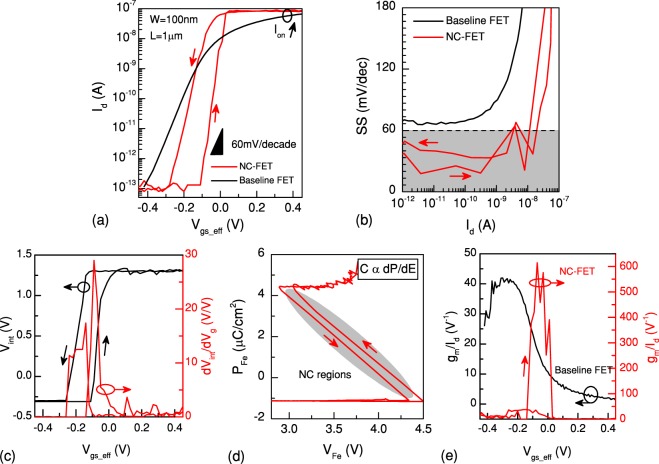
n-type NC-FET with a reduced hysteresis. (**a**) Performance of an n-type NC-FET with a small hysteresis of 150 mV and a swing below 30 mV/decade while V_*ds*_ is set to 100 mV (**b**). A steep *off*-to-*on* transition is realized in both positive and negative going branches of the drain current. (**c**) Internal voltage measurement shows a voltage gain of up to 10 V/V. (**d**) The extracted P-E curve of the ferroelectric shows a clear S-shape in a wide range of operation with a negligible hysteresis. (**e**) g_*m*_/I_*d*_ is also boosted and reached a factor of 600 V^−1^.

In consideration of (6), another NC-FET with a different baseline FET (W = 100 nm, L = 1 *μ*m) and a PZT capacitor of the same thickness and an area of 20 × 20 *μm*^2^ with better matching of capacitances is demonstrated in Fig. 4a. A reduced hysteresis of 150 mV is observed while the transistor is operating at a constant drain voltage i.e. 0.1 V. An SS below 30 mV/decade at 300 K is reliably achieved in both positive and negative going branches of the input transfer characteristic (see Fig. 4b). A possible reason for a steeper transition in the forward sweep comparing the reverse sweep can be explained by the asymmetry of the polarization (see Fig. 2 of supplementary materials). This occurs due to the difficulty of dipole flipping during the reverse sweep. Hence, dipoles partially get switched that reduces the impact of NC effect. The SS is below 30 mV/decade over four decades of the drain current. As a result, the effective gate voltage can be reduced by 50%, maintaining the same level of the output current. Figure 4c depicts the internal voltage and internal gain plots (V_*int*_ vs. V_*gs*_*eff*_ and dV_*int*_/dV_*g*_ vs. V_*gs*_*eff*_), illustrating a remarkable step-up conversion^5^. The extracted polarization characteristic of the series connected PZT capacitor (Fig. 4d) shows a clear S-shape polarization close to the ideal expectation of NC by LK equation. A small hysteresis is observed between the forward and reverse sweeps of the gate voltage. The current efficiency factor is also enhanced and reached a maximum value of about 600 V^−1^ (Fig. 4e). Although the performance boosting of the low hysteresis NC-FET in this last case is lower than the large hysteresis one, both analog and digital performances are remarkably enhanced comparing to the reference transistor. This means that a trade-off is required between the hysteretic behavior and the performance boosting that is caused by the NC of ferroelectric. Generally, considering that the SS can be expressed as *SS* = (60*mV*/*decade*).(1 + *C*_*MOS*_/*C*_*FE*_), the transistor transfer characteristic becomes steep as |*C*_*FE*_| gets close to *C*_*MOS*_. However, a value of |*C*_*FE*_| too close to *C*_*MOS*_ gives rise to the hysteretic behavior due to the instability of NC in the strong inversion regime^26^.

### p-type negative capacitance MOSFETs

**Figure 5:**
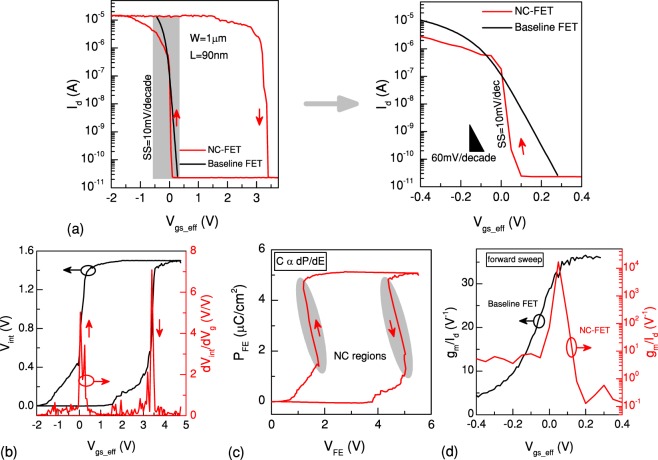
Hysteretic p-type NC-FET. (**a**) Transfer characteristic of a p-type NC-FET with a large hysteresis of 3 V (|*V*_*ds*_| = 900 mV) and a swing of 15 mV/decade over five decades of current. (**b**) An internal voltage gain greater than one is measured in both positive and negative going branches (**c**). Current efficiency factor is also enhanced, reaching a factor of 10^5^ V^−1^.

The impact of the same NC booster on p-type commercial MOSFETs and the hysteresis tuning with respect to (6) is reported and discussed. The drain-to-source voltage was set at −0.9 V in all measurements performed in this part, otherwise mentioned. Figure 5 depicts the input transfer characteristic of a p-type NC-FET (W = 1 *μ*m, L = 90 nm) using a PZT capacitor (40 × 40 *μm*^2^) as an NC booster. The gate voltage swept from +3 V to −3 V and returns back to the initial bias by reverse sweep. Using the NC booster, similar to n-type NC-FETs, the internal voltage is enhanced and reached values greater than the applied gate voltage, so that a steep *off*-to-*on* transition of 10 mV/decade is achieved over at least four orders of magnitude of the drain current (Fig. 5a). The NC condition is fulfilled in both forward and reverse sweeps so that a similar SS is demonstrated in both branches^28^. Due to the poor matching of capacitances, a large hysteresis of 3.5 V is obtained. Analyzing the internal electrode voltage (Fig. 5b) shows a considerable internal voltage amplification in the regions where the ferroelectric capacitor provides a clear S-shape negative slope of the polarization (Fig. 5c). An effective NC over a wide range of operation ensures a steep *off*-to-*on* transition together with a significantly enhanced g_*m*_/I_*d*_ FoM, reaching a peak of 10^5^ V^−1^ (Fig. 5d).

**Figure 6:**
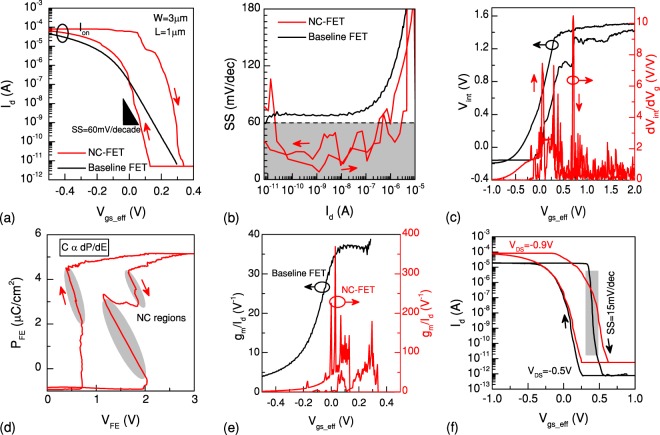
p-type NC-FET with a reduced hysteresis. (**a**) Input transfer characteristic of an NC-FET with a small hysteresis of 200 mV at |*V*_*ds*_| = 900 mV. (**b**) A sub-thermal swing well below 60 mV/decade is obtained. (**c**) Measurement of the internal node shows a significant voltage gain, having a peak of 10 V/V. (**d**) Polarization characteristic of the ferroelectric capacitor shows an effective NC in both branches. Two discrete NC regions are observable in the reverse sweep of the gate voltage due to the polycrystallinity of the ferroelectric film. (**e**) g_*m*_/I_*d*_ is considerably enhanced and reached a value of 400 V^−1^. (**f**) shows the impact of the drain-to-source electric field on the steepness and hysteresis of the NC-FET.

In a different structure, a p-type NC-FET with a better matching of capacitances and a small hysteresis is presented in Fig. 6a. A PZT capacitor with an area of 10 × 10 *μm*^2^ is connected to the gate of a p-MOSFET (W = 3 *μ*m, L = 1 *μ*m). A small hysteresis of 200 mV is achieved due to the proper capacitance matching. Figure 6b reports the SS vs. I_*d*_ plot which is well below the thermal limit of MOSFETs (down to 20 mV/decade) at 300 K. The internal node measurement confirms a voltage gain greater than 1 while having a peak of 10 V/V (Fig. 6c). The polarization vs. voltage plot of the PZT capacitor indicates a clear S-like curve in the positive going branch while it shows a different behavior in the reverse sweep. The ferroelectric performs two separate NC regions, demonstrating a zig-zag polarization characteristic. This mainly happens due to the fact that the polycrystalline PZT is showing two main polarization domains and a multi-domain ferroelectric capacitor in steady states cannot hold more than one negative capacitance domain at a time^18,29^. As a result, the manifested polarization characteristic of the multi-domain ferroelectric is different from the S-shaped curve which is expected for a single-domain ferroelectric (Fig. 6d). Therefore, each domain shows a separated NC region, also expected from dV_*int*_/dV_*g*_ vs. V_*gs*_*eff*_ curve where two individual peaks of the voltage amplification were clearly observed (see Fig. 6c). The equipotential connections by metal layers at the top and bottom surfaces of the ferroelectric capacitor are key parameters, preventing a multi-domain ferroelectric to exhibit the S-shaped P_*FE*_-V_*FE*_ (Q_*FE*_-V_*FE*_) curve expected from single domain ferroelectrics. Thus, a direct deposition of the ferroelectric on top of the gate oxide on semiconductor and reducing the area of the ferroelectric solve the addressed issue. Figure 6e illustrates the current efficiency enhancement with a maximum value of 400 V^−1^. The presented experimental results confirm the same impact and behavior of NC on both n- and p-type MOSFETs. Therefore, NC can be applied as an effective performance booster of CMOS with similar considerations for both types of transistors. Figure 6f investigates the impact of the drain-to-source voltage, |*V*_*ds*_|, on the input transfer characteristic of the same NC-FET. Besides the common effect of V_*ds*_ on the level of the drain current, it is evidenced that the NC-FET under higher lateral electric field performs a wider hysteresis window. In fact, the hysteretic behavior can be dramatically controlled by the drain voltage as the charge and MOS capacitances vary with V_*ds*_^28^. Moreover, the shape of the polarization curve is dictated by relative magnitudes of MOS and ferroelectric capacitances, meaning that the hysteresis can be tuned by V_*ds*_. Additionally, it is observed that the steepness of the *off*-to-*on* transition also changes with the drain voltage. An SS of 15 mV/decade is observed at a lower |*V*_*ds*_| of 0.5 V. In a ferroelectric MOS transistor, the ferroelectric polarization charge density and the channel charge density should match. Therefore, the operation point of the NC-FET is determined by the cross point of the P-E curve and the channel charge load line which depends on the drain voltage. Hence, changing the V_*ds*_ affects the operation point of the NC-FET and boosting effect of NC.

It is evidenced that the input transfer characteristic of NC-FETs with reduced hysteresis (both n- and p-type devices) is not as steep as one of the large hysteresis devices, also confirming the proposed theory that a trade-off is needed between the steepness and hysteretic behavior^27^. A ferroelectric capacitor that implies a too effective NC results in a large hysteresis together with a sharp transition. Although a super steep switching device is compelling, it is not appealing since the reduction of SS is accompanied by a remarkable hysteresis.

In conclusion, it has been shown that the negative capacitance effect can be effectively applied as a universal performance booster to enhance both digital and analog FoM of complementary MOS switches. The measured input transfer characteristics of advanced n- and p-type MOSFETs using PZT as the NC booster shows a steep subthreshold swing down to 10 mV/decade together with an enhanced efficiency factor up to 10^5^ V^−1^. The *on*-current over *off*-current ratio is improved and the overdrive is boosted up to 0.45 V. Therefore, the supply voltage can be reduced by 50%, maintaining the same performance. This is due to the fact that with the aid of a series connected negative capacitor (i.e., with the internal voltage amplification provided by the NC component of the PZT capacitor) the surface potential in MOS devices is increased beyond the applied gate voltage. It has been also demonstrated that the hysteretic behavior of NC-FETs can be tuned considering the proposed stability condition. Both n- and p-type NC-FETs with large (3–4.5 V) and reduced hysteresis (150–200 mV) are implemented, arguing that a trade-off is required between the steepness and hysteretic behavior of an NC-FET. The impact of the drain-to-source electric field on the boosting of NC is demonstrated and discussed, indicating that a lower lateral electric field in the channel results in a steeper *off*-to-*on* transition. Overall, this experimental study proposes and validates that a properly designed ferroelectric capacitor can be employed as a universal performance booster of CMOS transistors by offering an active gate stack, contributing to the signal amplification by improving the surface potential.

## Methods

As schematically shown in Fig. 2a, the experimental results are obtained by connecting an external PZT capacitor to the gate of a MOSFET. This external connection offers the flexibility to test different series combinations and tuning the hysteretic behavior. The employed structure can be modeled as a NC transistor with an intermediate metal layer between the ferroelectric and linear dielectrics of the gate stack. The presence of an intermediate metallic film ensures a uniform potential profile inside the ferroelectric, increasing the possibility of achieving a stabilized NC over a wide range of operation^30^. It has been previously reported that the NC state cannot be fully stabilized in a Metal-Ferroelectric-Metal-Insulator structure while the ferroelectric capacitor is leaky^31^. However, this is not a concern of this study due to the extremely low leakage current of the employed polycrystalline PZT capacitors.

High-performance commercial n- and p-type MOSFETs are employed as the baseline transistors. An MIM structure with 45 nm of polycrystalline Pb(Zr_43_, Ti_57_)O_3_ (PZT) is fabricated^32,33^. High-quality epitaxial ferroelectric layers are commonly considered suitable for NC devices due to the formation of a mono-domain state characterized by a single coercive field^6,31,34^. However, the typical behavior of poly-domain ferroelectrics can change dramatically by applying a repetitive voltage stress known as the training procedure of ferroelectrics^18^. This proposes that a poled ferroelectric layer behaves as a mono-domain like ferroelectric (see supplementary materials).

The measurement setup is explained in detail in the supplementary section. The source contact is grounded and a constant voltage is applied to the drain contact. The gate voltage is ramped by 5 mV steps, applied for 500 *μ*Sec and hold for the same time. The measured transfer characteristics of NC-FETs using a different sample, hold, and integration times through the possible range of the parameter analyzer showed that the reported results are stable and irrespective of complex measurement dynamics. Additionally, the available points in the negative slope region of the extracted polarization characteristic confirm that the steep switching of transistors corresponds to the NC of the ferroelectric capacitor and it is not an artifact of measurement. The output transfer characteristic of NC-FETs is not discussed in this paper due to the fact that no considerable NC effect can be observed at a constant gate voltage. As it is explained in detail in the supplementary section, a sufficiently large change of electric field is required in order for a ferroelectric material to perform NC effect. Therefore, at a constant gate voltage, the ferroelectric capacitor just acts as a positive capacitor and the Fe-FET operates as a conventional transistor.

## Supplementary information

supplementary information
