# Supplementary material for: Negative Capacitance as Universal Digital and Analog Performance Booster for Complementary MOS Transistors
Source: Sci Rep. 2019 Jun 24;9:9105. doi: 10.1038/s41598-019-45628-8 (PMC6591349; doi:10.1038/s41598-019-45628-8)
Supplement: Supplementary file 1 — supplementary information [file 41598_2019_45628_MOESM1_ESM.pdf]

Negative Capacitance as Universal Digital and Analog Performance Booster for Complementary MOS Transistors

Ali Saeidi[1] , Farzan Jazaeri[1], Igor Stolichnov[1], Christian C. Enz[1], and Adrian M. Ionescu[1]

[1]Ecole Polytechnique Federale de Lausanne, Lausanne, Switzerland

Fabrication and Characterization of PZT

For this experiment, 46±3 nm of $Pb(Zr_{43},Ti_{57})O_3$ (PZT) ferroelectric film has been grown via the chemical solution deposition root (REF) on a Pt-coated silicon wafer. The stack of Pt(100 nm)/TiO_2(30 nm) has been sputtered on SiO_2(500 nm)/Si wafer at 300 °C. The PZT film consisted of tetragonal ferroelectric phase with the predominant (100) orientation, with virtually no inclusion of any secondary non-ferroelectric phase like pyrochlore, as confirmed by XRD theta-2theta scans. The polycrystalline PZT film had a dense columnar grain structure with the grain size of 200±100 nm. Pt top electrodes were deposited on PZT film by sputtering and post-annealed at 550 °C in an oxygen atmosphere in order to remove the sputtering damage at the Pt/PZT top interface.

The ferroelectric capacitors exhibited the dielectric properties typical for high-quality ferroelectric PZT layers of this type as it is illustrated in Figure 1 and 2. Specifically, the polarization hysteresis loop measured at 1 KHz showed the remanent polarization of 25±3 $\mu C/cm^2$ and coercive fields of +80/-300 KV/cm, with the polarization imprint that favors the top-to-bottom polarization direction. Quasi-static C-V measurements show the hysteretic behavior of the dielectric constant with the maximum values of 600-700 near the coercive voltage. [1].

It is remarkable that the negative capacitance (NC) effect has been reached using a chemical-solution-deposited polycrystalline PZT film. Generally, the high-quality epitaxial ferroelectric layers are considered suitable for NC devices [2] as they are more likely to form a mono-domain state characterized by a single coercive field. This is in contrast with the typical behavior of the polycrystalline films, which tend to form complicated poly-domain patterns with a broad distribution of nucleation energies and coercive fields. In the present study, we show that this behavior can be changed dramatically by applying a repetitive bipolar voltage stress known as the training procedure of ferroelectrics. In this regard, we have measured the local polarization switching using the off-resonance Piezoelectric Force Microscopy (PFM).

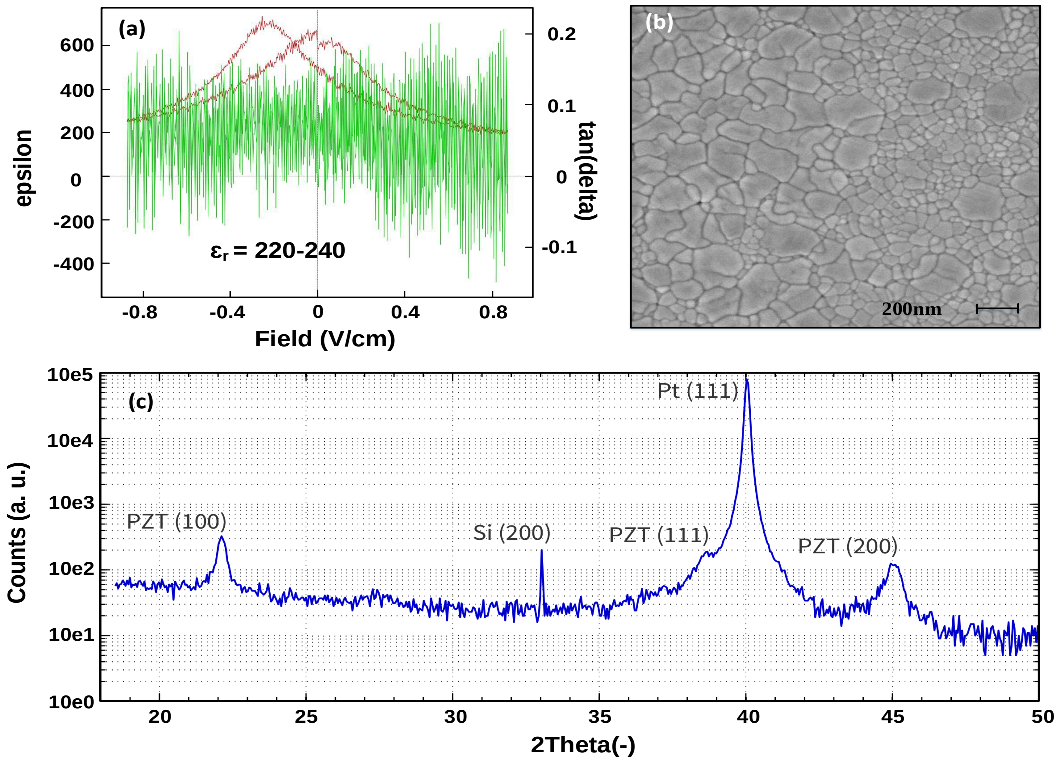

Figure 1. The electrical and physical characterization of PZT ferroelectric thin film. 46 nm of PZT with 43/57 (Zr/Ti) ratio is deposited on a TiO_2(2 nm)/Pt(100 nm)/TiO_2/Ti/SiO_2/Si substrate by employing the sputtering technique. Pt top electrode is deposited at room temperature and patterned by shadow masking. The film polarization, permittivity, and the phase angle of the capacitance measurement hysteresis loops regarding the applied voltage (electric field) on the ferroelectric layer are depicted in (a). The relative permittivity of the PZT thin film is 220-240. The SEM analysis (b) and XRD 2-theta profile (c) of the PZT thin film illustrate that the film is polycrystalline (see microstructures in SEM image) and textured 111-oriented, which is the most commonly used orientation.

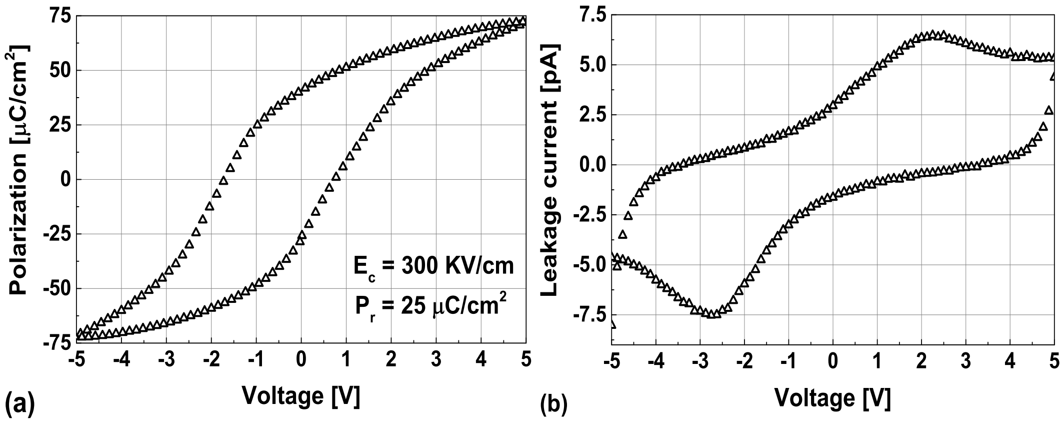

Figure 2. The polarization (a) and current (b) hysteresis loops of the fabricated PZT capacitor. The extracted values of the film coercive field and remanent polarization is around 260 KV/cm and 25 $\mu C/cm^2$ respectively.

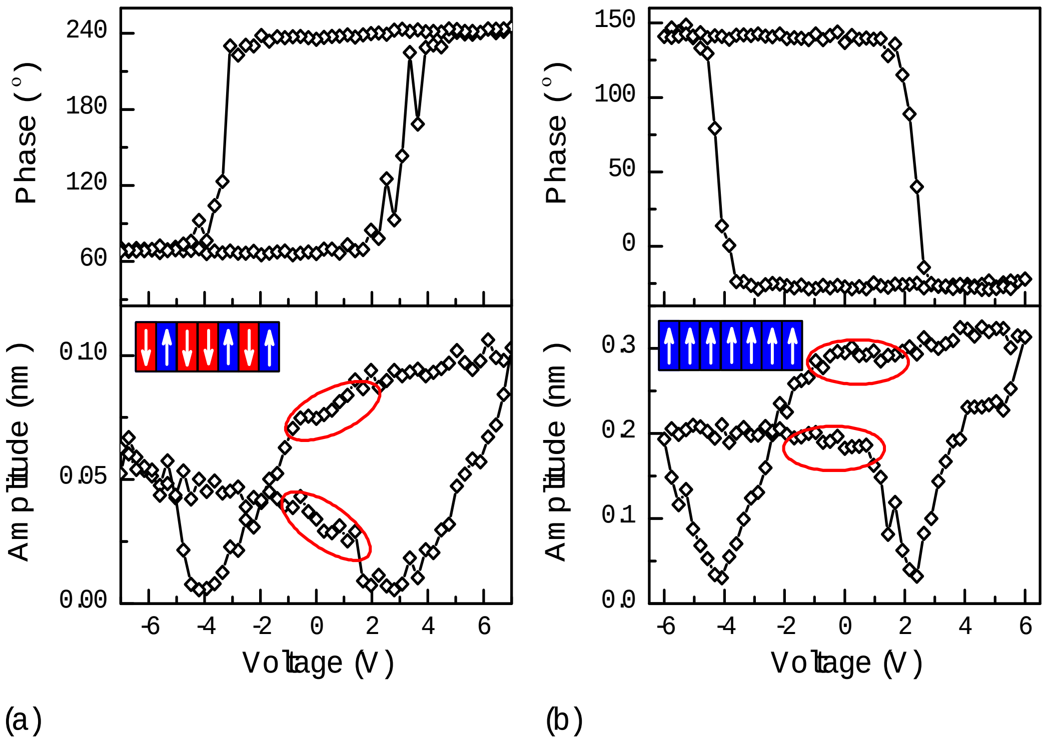

Figure 3. Piezoelectric response of the PZT thin film. The results are measured before (a) and after (b) the ferroelectric training procedure. This behavior suggests that the poled ferroelectric layer approaches the mono-domain behavior and doesn't switch at least at low DC voltages up to 2 V.

The technique has been enhanced for probing extremely weak electromechanical coupling with an outstanding sensitivity < 0.1 pm. Piezoelectric loops measured through the top electrode of the PZT capacitor after 20 cycles at ± 7 V show sharp switching and nearly constant piezoelectric response. The results are presented in Figure 3 for the polycrystalline PZT before (a) and after (b) the training procedure in terms of the amplitude and phase of the measured signal. This behavior suggests that the poled ferroelectric layer approaches the mono-domain-like behavior. Note that the piezoelectric loops collected on the as-fabricated capacitor without any training reveal different behavior typical for region-by-region poly-domain switching expected from a polycrystalline film. The demonstration of NC effect using a polycrystalline ferroelectric layer constitutes a significant step towards the integration of NC gates in CMOS technology. In fact, fabrication of epitaxial perovskite layers on silicon is an extremely challenging task, whereas polycrystalline ferroelectrics like PZT can be integrated, as shown in previous reports [3, 4].

Electrical characterization of NC-FETs

As schematically shown in Figure 4a, the experimental results are obtained by connecting an external PZT capacitor to the gate of a MOSFET. This external

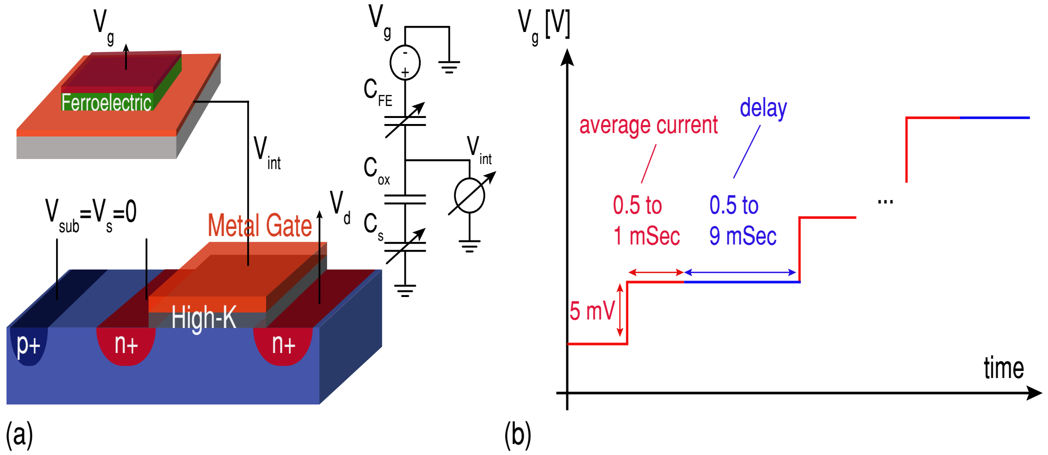

Figure 4. Measurement setup (a) and the ramp of the gate voltage sweep (b) for the NC-FET electrical characterization.

connection offers the flexibility of testing tens to hundreds of PZT capacitor and MOSFET configurations to tune the hysteretic behavior of the resulting NC-FET. The source contact is grounded and a constant voltage is applied to the drain contact. The gate voltage is ramped, in correspondence to the schematic diagram of Figure 4b, while the internal contact is probed. It has been observed that the achieved results of this work are repeatable and almost identical for different measurement conditions, as demonstrated in Figure 4b.

Impact of Negative Capacitance on the output transfer characteristic

As it is noted in the main manuscript, the concept of ferroelectric's negative capacitance can be understood by considering the free energy of this sort of materials. A ferroelectric material is traditionally modeled using the double well energy landscape. In equilibrium, the ferroelectric resides in one of the wells and provide spontaneous polarization. The capacitance of the ferroelectric is positive around these wells as can be understood by the curvature of the energy landscape. However, when the dipole switches from one stable polarization state to the other one, it passes through a region where the curvature of the free energy landscape is negative and hence, shows an effective NC (Figure 5). Clearly, the dipoles cannot even partially switch by sweeping the drain voltage while the gate voltage is constant and hence, no considerable impact due to the negative capacitance effect can be observed. The sweeping of the drain voltage does not provide any considerable change in the vertical electric field inside the ferroelectric. The ferroelectric dipoles do not change their stable state and the capacitor acts as a linear dielectric. In this case, the structure works as a capacitive voltage divider between V_{gs} and V_{int} and the transistor works as a conventional MOSFET with reduced performance.

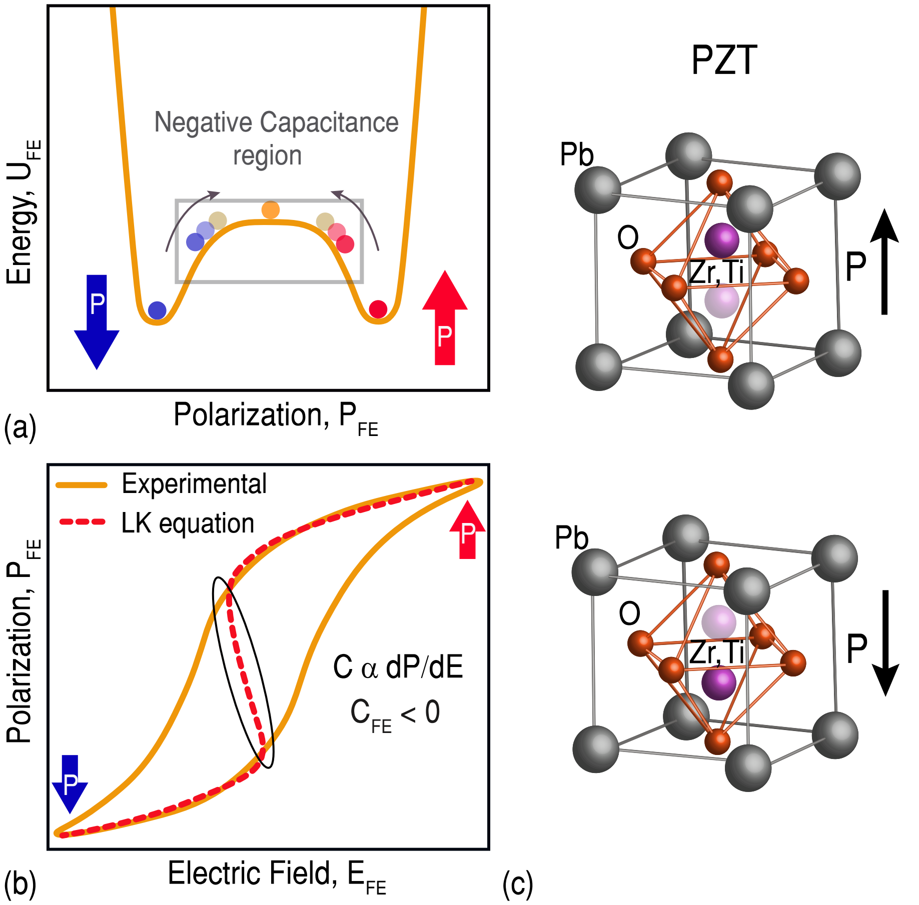

Figure 5. Negative capacitance in ferroelectric materials. (a) Energy density function of a ferroelectric capacitor in equilibrium, showing an effective NC while switching from one stable polarization state to the other one. (b) According to Landau model, a ferroelectric material can perform NC effect while dipoles change direction. (c) Crystal structure of PZT and its two stable polarization states.

References

[1] SR Gurkovich and JB Blum. Crystallization of amorphous lead-titanate prepared by a sol-gel process. *Ferroelectrics*, 62(1):189–194, 1985.

[2] Pavlo Zubko, Jacek C Wojdeł, Marios Hadjimichael, Stéphanie Fernandez-Pena, Anaïs Sené, Igor Luk'yanchuk, Jean-Marc Triscone, and Jorge Íñiguez. Negative capacitance in multidomain ferroelectric superlattices. *Nature*, 2016.

[3] S Dasgupta, A Rajashekhar, K Majumdar, N Agrawal, A Razavieh, S Trolier-Mckinstry, and S Datta. Sub-kt/q switching in strong inversion in $PbZr_{0.52}Ti_{0.48}O_3$ gated negative capacitance FETs. *IEEE Journal on Exploratory Solid-State Computational Devices and Circuits*, 1:43–48, 2015.

[4] Daniel JR Appleby, Nikhil K Ponon, Kelvin SK Kwa, Bin Zou, Peter K Petrov, Tianle Wang, Neil M Alford, and Anthony O'Neill. Experimental observation of negative capacitance in ferroelectrics at room temperature. *Nano letters*, 14(7):3864–3868, 2014.
